# Carcinome basocellulaire de la face: à propos de quatre cas rapportés à Madagascar

**DOI:** 10.11604/pamj.2015.22.97.7154

**Published:** 2015-10-02

**Authors:** Rex Mario Razafindrakoto, Mananjara Nandrianina Razafindranaivo, Mahamad Rojovolaarivony Schammirah, Rado Randriamboavonjy

**Affiliations:** 1 Service d'Oto-Rhino-Laryngologie et de Chirurgie Cervico-Faciale, Centre Hospitalier Universitaire d'Andohatapenaka, Antananarivo, Madagascar; 2 Faculté de Médecine Humaine d'Antananarivo, Antananarivo, Madagascar

**Keywords:** Anatomie pathologique, carcinome basocellulaire, métastase, traitement, anatomopathology, basal cell carcinoma, metastasis, treatment

## Abstract

Les carcinomes basocellulaires, fréquemment rencontrés dans la race blanche, sont plus rares chez les sujets de race noire. Les zones exposées de la tête sont des sites préférentiels, et une intense exposition aux rayons solaires ultraviolets a été évoquée dans leur étiopathogénie. Les métastases sont exceptionnelles. Les objectifs ont été de démontrer l'existence de carcinomes basocellulaires à Madagascar et d'en évaluer la prise en charge. Les auteurs ont rapporté quatre cas de carcinomes basocellulaires faciaux vus au service d'Oto-rhino-laryngologie du Centre Hospitalier Universitaire d'Antananarivo, avec deux hommes et deux femmes, âgés entre 46 et 70 ans (âge moyen= 53,5 ans). Une exérèse chirurgicale a été pratiquée chez trois patients tandis qu'un patient albinos a été traité par radiothérapie. L’épidémiologie, l’étiologie, l'anatomie pathologique et le traitement des carcinomes basocellulaires de la face ont été discutées à travers une revue de la littérature.

## Introduction

Les carcinomes basocellulaires (CBC) constituent le cancer cutané le plus fréquent de la population blanche [1]. Ils sont plus rarement retrouvés chez la race noire [2, 3]. Les objectifs de cette étude ont été de rapporter des cas de CBC de la face chez les Malgaches, et d'essayer de faire ressortir un protocole thérapeutique à adopter face à cette pathologie.

## Patient et observation

**Figure 1:**
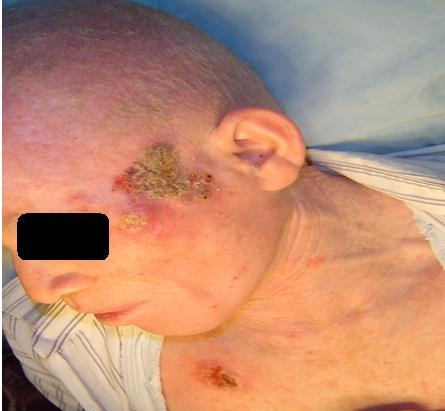
Carcinome basocellulaire superficiel localisé au niveau de la face, du thorax et des mains chez un sujet albinos

**Figure 2:**
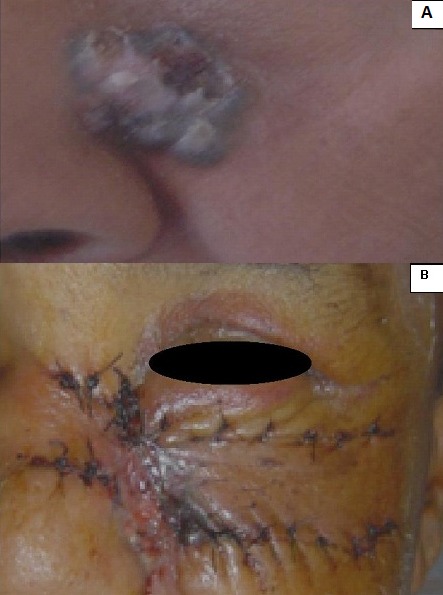
A) Carcinome basocellulaire nodulaire infra-orbitaire gauche, avant exérèse chirurgicale; B) Même patient qu'avec la figure 2A, après exérèse chirurgicale

**Figure 3:**
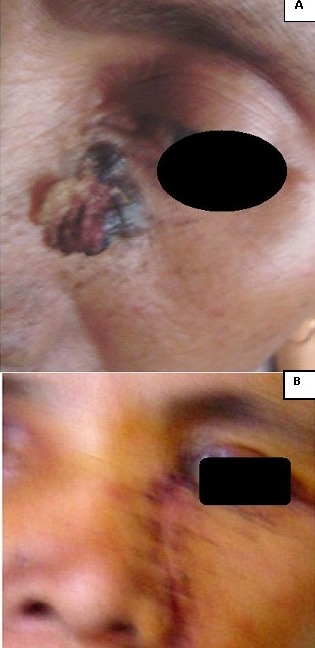
A) Carcinome basocellulaire nodulaire infra-orbitaire gauche; B) Même patiente qu'avec la figure 3A. Pas de récidive trois ans après l'exérèse chirurgicale.

**Figure 4:**
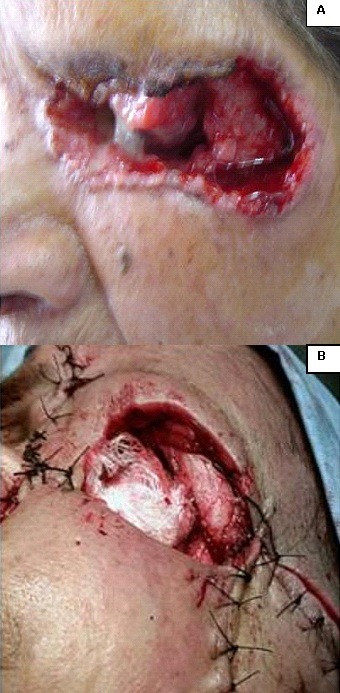
A) Carcinome basocellulaire orbitaire gauche avec aspect typique de morsure ou «*ulcer rodens*»; B) Même patiente qu'avec la figure 4A. Exentération et reconstruction difficile par confection de grands lambeaux

De Janvier 2009 à Mai 2012, sur 18.500 consultants dénombrés au service d'Oto-rhino-laryngologie du Centre Hospitalier Universitaire d'Antananarivo, Hôpital Universitaire Joseph Ravoahangy Andrianavalona, quatre ont été atteints d'un CBC de la face. Dans un tableau récapitulatif (Tableau 1) est détaillée la répartition de nos patients selon le genre, l’âge et leurs phénotypes qui ont rappelé l'origine lointaine du peuplement Malgache. Celle-ci a été Arabe, Africaine, ou Asiatique (Indonésienne et Malaisienne). Il y a eu un malade albinos (Figure 1). Le tableau 1 a précisé également les professions de nos patients, lesquels se sont déroulés habituellement au grand air (agricultrices, pisciculteur et éleveur), ainsi que les localisations anatomiques des lésions. Chez deux patients, une tumeur ferme, en relief, bien limitée, lisse, ulcérée par endroits a été retrouvée, permettant de faire suspecter le diagnostic de CBC nodulaire (Figure 2A, Figure 3A). Chez le patient albinos, les lésions ont été recouvertes de squames et de croûtes, orientant vers le diagnostic de CBC superficiel (Figure 1). Une vaste ulcération orbitaire, avec destruction tissulaire importante, donnant un aspect typique de morsure ou *ulcer rodens*, a été retrouvée chez une patiente (Figure 4A). Dans tous les cas, l'examen anatomopathologique de prélèvements biopsiques tumoraux a conclu à un CBC. Un bilan d'extension comprenant une radiographie pulmonaire et une échographie abdominale a été sans particularités pour tous nos malades. Une exérèse chirurgicale dépassant d'un centimètre les rebords de la tumeur a été réalisée chez trois patients, avec confection de grands lambeaux, non pédiculés (Figure 2B, Figure 4B). Une exentération a été nécessaire pour une patiente (Figure 4B). Le résultat esthétique postopératoire a été acceptable chez deux patients (Figure 2B, Figure 3B), mais moins bon chez la patiente qui a présenté l’*ulcer rodens* (Figure 4B). Le malade albinos a été traité par radiothérapie, amenant une disparition des lésions. L'examen anatomopathologique des pièces opératoires a confirmé une exérèse tumorale complète, avec marges saines. Après un recul de deux à trois ans, aucun patient n'a présenté ni récidive ni métastase.

**Tableau 1:** Récapitulation de nos observations

Observations	Genre	Age	Professions	Phénotypes des patients	Localisation des lésions
1	masculin	51 ans	éleveur	Albinos	face, thorax et mains
2	masculin	47 ans	pisciculteur	Africain	infra-orbitaire gauche
3	féminin	46 ans	agricultrice	Asiatique	infra-orbitaire gauche
4	féminin	70 ans	agricultrice	Asiatique	orbitaire gauche

Il y a eu deux patients de phénotype Asiatique (cheveux lisses, teint clair), un de phénotype Africain (cheveux crépus, teint halé, nez épaté et lèvres épaisses), et un albinos. Les âges extrêmes ont été de 46 et 70 ans, et l’âge moyen de 53,5 ans

## Discussion

L'incidence des CBC est de 110/100.000 habitants avec la race blanche, particulièrement élevée en Australie où elle a été évaluée à 788 cas pour 100.000 habitants et par an [1]. Il n'existe pas de travaux approfondis et définitifs sur la prévalence des cancers cutanés au sein des populations noires Africaines. Les carcinomes épidermoïdes (ex-spino-cellulaires), diagnostic différentiel des CBC, l'emporteraient cependant en fréquence sur les basocellulaires [3, 4]. Nos patients ont été de phénotypes Africain ou Asiatique comme le sont les Malgaches (Tableau 1). Nos patients, quatre vus en deux ans et demi, ont été découverts de manière sporadique. Comparée à la littérature Africaine consultée [3, 5], où les cas rapportés ont été au nombre d'un ou deux, les nôtres ont été plus nombreux. Sur une série de 181 CBC, Hakverdi a noté une prédominance de l'atteinte masculine (101 du genre masculin contre 80 du genre féminin), alors que les deux genres ont été également représentés dans notre série, cependant plus petite que celle d'Hakverdi [1]. Cet auteur a rapporté un âge moyen de 64,11 ans chez les hommes contre 59,33 chez les femmes, alors que l’âge moyen de nos patients, les deux genres confondus, a été de 53,5 ans (Tableau 1) [1]. Les cancers cutanés seraient liés à l'exposition prolongée et continue aux rayons ultraviolets solaires, surtout lors des activités professionnelles en plein air, comme celles exercées par nos patients (agricultrices, éleveur et pisciculteur) (Tableau 1) [3]. La forte pigmentation cutanée des individus de race noire les protègerait contre les carcinomes cutanés. L'albinisme constituerait dans ce contexte un facteur de risque [3]. Chez les albinos, la tumeur est plus rapidement évolutive, et l’âge de survenue plus précoce que chez les patients non albinos [2]. Notre patient albinos a été âgé de 51 ans et a présenté des lésions multiples (faciales, thoraciques et membres) (Figure 1). La nature de la cellule pluripotentielle à l'origine des CBC est restée inconnue [3]. Les CBC sembleraient survenir de *novo*, sans lésion préalable, mais la transformation d'une kératose solaire en CBC serait possible [3]. L'une des preuves du rôle primordial étiologique joué par le rayonnement solaire est la localisation préférentielle des CBC sur certains sites anatomiques plus exposés, comme la face- incluant les paupières-, le cou, les auricules ou le cuir chevelu, où sont retrouvées les deux-tiers des CBC [3]. Ahmad a montré une localisation des CBC départagée de façon identique entre des zones plus exposées (front, joues et pyramide nasale) et des zones moins exposées (auricules et cuir chevelu) [6].

Une biopsie tumorale suivie d'un examen anatomopathologique doit être effectuée pour confirmer le diagnostic, recherchant une prolifération de kératinocytes anormaux, avec des cellules basaloïdes agencées de façon variable, en travées, en lobules ou en nodules, avec une disposition palissadique des noyaux en périphérie [7]. Confirmé par le bilan d'extension n'ayant révélé aucune anomalie chez nos patients, les CBC provoquent exceptionnellement des métastases, même à une phase avancée [2, 8], ce qui les différencie des carcinomes épidermoïdes qui donnent des métastases. Celles-ci sont rencontrées dans 28 cas pour 10. 000 patients atteints de CBC [3]. Un CBC de quelques millimètres peut être pris en charge par le dermatologue, qui ne dispose pas du plateau technique nécessaire pour l'ablation de tumeurs évoluées. Des excisions par coupes horizontales sont effectuées dans la technique micrographique de Mohs. Les CBC traités par Galimberti par cette technique ont été de petit volume, le défect cutané ayant mesuré en moyenne 0,86 centimètre carré [7]. La chirurgie micrographique de Mohs est inapplicable face à nos grosses tumeurs. Dans les CBC ayant acquis des dimensions importantes- comme les nôtres-, l'ablation par une chirurgie conventionnelle est la méthode thérapeutique de choix [3]. L'exérèse doit être carcinologique, avec des marges de résection saines, et nécessite une maîtrise de la technique des lambeaux locorégionaux [5]. L’état évolué de nos tumeurs a rendu nécessaire une marge d'exérèse supérieure à dix millimètres, obligeant à un geste plastique difficile avec de grands lambeaux (Figure 2B, Figure 4B). Ceux-ci ont heureusement conservé une bonne vitalité grâce à la bonne vascularisation des téguments de la face. Une plastie en V-Y peut être pratiquée dans les CBC très larges et évolués [8]. Même après exérèse large, les récidives de CBC sont possibles, notamment pour ceux qui ont dépassé deux centimètres [9]. Cela a imposé une surveillance post-chirurgicale attentive et prolongée de nos patients chez lesquels le recul a été de deux à trois ans, sans récidive. Les récidives sont plus fréquentes chez les sujets ayant présenté une localisation multiple [9, 10]. Un risque élevé de récidive existe donc chez notre patient albinos, nous incitant à une surveillance attentive chez ce sujet. La radiothérapie constitue une alternative à la chirurgie, (comme cela a été effectué chez le patient albinos) (Figure 1), mais peut aussi être utilisée à titre complémentaire [3]. La chimiothérapie n'est indiquée qu'en cas de tumeur très évoluée et inopérable [10]. Mis à part pour le patient albinos, aucun traitement oncologique n'a été prescrit pour nos malades. Cette option thérapeutique a été réservée pour une éventuelle récidive tumorale. Les crèmes antisolaires ont été proposées pour prévenir l'apparition des CBC [10]. Comme elles ne sont pas d'utilisation courante dans notre pays, nous recommandons le port de chapeaux et de vêtements à manches longues chez ceux qui travaillent au soleil et chez les patients albinos, impliquant une éducation sanitaire.

## Conclusion

Si le carcinome basocellulaire est le plus fréquent des cancers cutanés dans la race blanche, à travers nos quatre observations, nous concluons que cette pathologie existe chez les Malgaches, aux origines Arabes, Africaines et Asiatiques. La chirurgie, conventionnelle ou micrographique, et/ou la radio-chimiothérapie constituent la base du traitement. Des recherches ultérieures pourront déterminer l’épidémiologie des carcinomes basocellulaires à Madagascar, où les tumeurs peuvent encore prendre des proportions considérables, du fait d'un diagnostic souvent tardif.
